# Dr. Gellerstedt on Tubercular Phthisis

**Published:** 1847-04

**Authors:** 


### Art. VIII.

*Bidrag till den Tuberkulösa Lungsoten's Nosographi och Pathologi.* Af. Pehr Erik Gellerstedt, Med. Doctor, &c., och Sjukhus Läkare vid Kongl. Allm. Garnizons Sjukhuset i Stockholm.—*Stockholm,* 1844.

*Contributions to the Nosography and Pathology of Tubercular Phthisis.* By Peter Erik Gellerstedt, M.D., and Physician to the Royal Garrison Hospital in Stockholm.—*Stockholm,* 1844. 8vo, pp. 127.

We have recently had the pleasure of introducing to the notice of our readers the writings of a Swedish physician, who, though as yet comparatively a young man, has obtained considerable eminence as an acute and diligent observer in his own country. In the Clinical Reports of Dr. Huss, to which we allude, we had occasion to regret that we could not present to our readers the opinions of that author upon many important diseases, from the circumstance of his having treated of them in other of his annual volumes, to which unfortunately we had not access. The present work, in a manner, supplies a part of this deficiency, by laying before us the opinions and experience of one placed in the most favorable position to observe, and who is, if we may judge from the work before us, fully competent to the task. Our knowledge of phthisis, as it appears in various countries of Europe, has of late years been very much extended, but we have not seen a single recent work, even from the laborious press of Germany, that gave any account of this disease as it exists in the Scandinavian peninsula. Nor must we look upon the present essay as completely filling up the hiatus we complain of; for it pretends only to be the result of observations made in the Military Hospital at Stockholm, where soldiers alone are admitted. The general results of Dr. Gellerstedt's researches cannot therefore be of much avail in the statistical history of phthisis. But our author, at the very beginning, expressly assures us that it is not the general history of phthisis which he pretends to write, but merely to illustrate certain symptoms and features of the disease as it has occurred in his own practice.

Dr. Gellerstedt believes that phthisis in Sweden is upon the increase. The fact, if a fact, may perhaps be accounted for on the supposition that more luxurious habits are beginning to invade the stern regions of the north; but it is more probable that, as the means of diagnosis are now so much improved, the disease is only more readily detected. In the garrison at Stockholm, consisting of about 3500 men, the average mortality is about 80 deaths per annum, and of these 28 die from phthisis, while many more, affected with the same malady, are dismissed as incurable from the regiment, and return to die in their own homes. This gives a per centage upon the total deaths from all diseases of 35 per cent. in favour of phthisis.

The researches of our author during his five years of service in the hospital, have been chiefly directed to the pathology of the disease, and especially to the earliest commencement of the organic changes in the lungs. This, it is acknowledged by all, is the most interesting and important point in its whole history, nor can we say, in spite of the elaborate researches of Louis, Fournet, and others, that the subject is in any way exhausted.

In a brief preface, our author gives us, as preliminary to the other matters, the result of his own researches on the intimate structure of the lungs. He differs from Reisseisen and others as to the mode of termination of the bronchi. That author believed that each ultimate division of a bronchus was terminated by a single cell; Dr. Gellerstedt, on the contrary, inclines to the opinion of Dr. Hodgkin, that the bronchi diminish till their diameter is equal to that of two air-cells, and then really terminate. For, on slicing dried portions of the pulmonary parenchyma, and placing them under the microscope, numerous air-cells may be seen opening directly into the parietes of the bronchial tube, which tube cannot be traced beyond that point. In a word, as our author vividly expresses it, these air-cells line the walls of the ultimate bronchial divisions (*tapetsera dess väggar*). After briefly analysing the rest of Reisseisen's well known descriptions, Dr. Gellerstedt states, that he has recently, for very fine microscopical injections, employed blood deprived of its fibrine:

"I inject this," says he, "through the pulmonary artery, till it pours out from the opening of the pulmonary veins; I then tie both vessels, and, inflating the lungs, place a ligature on the bronchi, to prevent the escape of the air. After this, the whole mass is sunk in a vessel containing dilute sulphuric acid, in which it is allowed to lie for one or two hours, until the colouring matter of the blood is entirely coagulated throughout the whole lung. It is then taken out and slowly dried, until it becomes sufficiently hard to allow of its being cut into very thin slices."

The lining membrane of the ultimate division of the bronchi scarcely, he thinks, can claim the appellation of a mucous membrane, as the muciparous glands are wholly wanting; nor is the fine villous coat, so characteristic of that tissue, any longer to be found.

From these short, but interesting, notices of the pulmonary parenchyma, our author passes to the more immediate subject of tubercular degeneration, and first discusses the interesting point of the nature of *miliary tubercle.* He admits with Laennec, that the gray semitransparent granulations are of tubercular origin, but he has not generally met with a yellowish softer point in the centre of these small bodies. Dr. Gellerstedt has, however, frequently found portions of the miliary tubercle to be of a different colour from the appearance it generally presents, but he considers this to result rather from *engaged portions of the parenchyma of the lungs,* than from any change operated in the granulations themselves. Andral and Lombard's opinion that these granulations are the result of a croup-like exudation into separate air-vesicles, he does not consider to be supported by microscopical examination, for the granulations are too large to be contained in single air-vesicles, and, besides, they are seen to contain numerous engaged fragments of the pulmonary parenchyma, which, were their theory correct, could not be the case.

*Crude tubercle,* properly so called, is distinguished from the gray granulations by its opacity and by its yellow colour. It has not, observes Dr. Gellerstedt, the well-rounded form that generally distinguishes the miliary variety. Our author describes also a round isolated tubercle of not very frequent occurrence, which is of a firm wax-like consistence, and often attains, unaltered, the size of a hazel nut. It is surrounded with a firm cyst, and is generally found isolated in the lungs: it has been observed by Dr. Gellerstedt in 24 cases, in most of which the patients had died of tubercular phthisis.

*Tubercular infiltration* of the lungs is minutely described. Dr. Gellerstedt admits of two varieties of this alteration: the one he terms isolated tubercular infiltration (*insulär tuberkel infiltration*); the other is that general form, so well delineated by Rokitansky. We do not remember to have seen any better description of the former variety, and we therefore introduce it here from Dr. Gellerstedt's work:

"Insular tubercular infiltration occurs in masses, from a pea to a walnut in size, and is of a blueish-gray colour, with a smooth and almost fatty aspect when cut. The tissue of the lungs, when this occurs, has entirely lost its accustomed porous appearance, without assuming the character of hepatization, or that given by aggregated miliary tubercles. At the edge of these gray masses we observe many small yellowish-white points of a looser consistence, and which consist of softened tubercular matter. These points are sometimes so abundant that they surround the gray infiltrated masses as with a ring. It is rare that they are to be found within the mass itself."

Insular or isolated infiltration of the lung occurred in 17 cases out of 119, or in 14 per cent. of those who died of phthisis, and in 11 per cent. of those whose death was occasioned by other maladies.

*Caverns* or excavations in the lungs occurred, as might be expected, very frequently, but in only 4 instances out of 119 did they preponderate, or appear only in the *lower* lobes of the lungs. The larger bronchi are generally described as being abruptly cut off at their entrance into these excavations, but Dr. Gellerstedt states that a slightly swollen ring is usually formed around the orifice by the mucous membrane lining the air-passages. He has only once observed a *single cavern* in patients who died of phthisis, but he has met with single excavations seven times in those who had succumbed to other maladies. Dilatations of the bronchi were frequently observed, and in one instance, where the patient did not die of phthisis, so large a cavity had been formed in this way, that it abutted upon the pleura, which was adherent to the ribs, and threatened to form a communication with the external parietes. The bronchi, too, are frequently compressed in phthisis; Dr. Gellerstedt has traced a bronchus thus flattened, and forming a compact ligament of two lines in diameter, till it ended in a double-headed mass containing a calcareous concretion.

*Ulcers in the larynx, &c.* In speaking of ulcerations of the trachea, Dr. Gellerstedt observes, that the mucous membrane surrounding these ulcers is frequently covered with a white albuminous layer, similar to that described by Bretonneau under the name of diphtheritis. True tubercle or tubercular matter he has never met with in the *trachea*. These ulcerations appear to supervene at an advanced stage of the disease, for out of 45 cases in which they were observed, 42 were so far advanced, that caverns already existed in the lungs. The ulcerations in the larynx, so common in phthisis, are, however, believed by Dr. Gellerstedt to be truly of tubercular origin, while those of the trachea here described, he regards, with Louis, as of an aphthous nature, arising from the constant irritation of the cough.

In two instances the pericardium contained tubercular matter in the form of calcareous concretions. One of these was about the size of a walnut, and consisted of a cyst with thick walls, containing a substance in consistence and appearance exactly resembling whitewash.

*The intestinal canal* presented very frequent lesions, tubercular deposit having occurred there in 97 cases out of 119. It appeared in various forms: *a.* As *granulations;* in size and consistence resembling miliary tubercles, but differing from these in their colour, being whitish-yellow and opaque. They were not confined to the solitary or agminated glands, though they were rarely met with in the large intestines, their seat being almost always in the submucous cellular tissue of the ileum. *b.* As *ulcerations;* occurring in the usual form: but we cannot think our author justified in separating these two varieties, as it is most probable that tubercles always form where ulcerations are found, but have subsequently softened and have been carried away.

Tubercular ulceration of the *stomach* is of rare occurrence; Dr. Gellerstedt has observed this alteration not less than five times in 119 cases of death from phthisis. It remains, we think, to be proved that these ulcerations were really tubercular.

*The mesenteric glands* presented no other form of disease than tubercular infiltration, and were less frequently affected than the intestines, in the proportion of 44 to 97. Our author met with but five cases out of 46 in which the mesenteric and the bronchial glands were simultaneously tuberculous. He rarely observed the fatty degeneration of the liver so accurately described by Louis.

It was only in two instances out of all his dissections that Dr. Gellerstedt found tubercular matter in other parts of the body when it did not exist in the lungs. In both these cases its site was the bronchial glands, and in one a cyst full of tubercular matter existed in the pericardium.

It will be thus seen that the proportion of cases of tubercular degeneration of the intestinal canal is much greater in Dr. Gellerstedt's reports than in those of Louis or of Lombard. Our author accounts for this, by supposing that the intestinal canal is more frequently affected in Stockholm than in Geneva or Paris, yet the statistics of typhoid fever give a contrary result.

Tubercles of the lungs accompanied Bright's disease of the kidney, in the proportion of 7 per cent.

From these minute but interesting results of his post-mortem examinations, Dr. Gellerstedt next passes to the obscure subject of the pathology of phthisis, and attempts to lay before us a full history of the origin and development of tubercle. With the physiologists of the present day our author believes tubercle to commence like other tissues, healthy or diseased, as a cytoblastema, yet with Vogel, he believes that something more is wanting to produce tubercular matter, some vitiation of that vital influence that develops the cytoblastema into healthy tissue, but which, when weakened in any way, causes the same cytoblast to pass into pseudo-organization, such as tubercle or schirrus. The germination (if we may use the term) of a cytoblast, is the same for all the tissues of the body, but the cells which are developed as nuclei in the original vesicle, first show the different tissues to which they are to be adapted, as a chemical [?]

change takes place in their parietes, and under a vitiated influence they are altered sometimes to tubercle, which in its highest stage of development consists of cells, with more or less of granular matter lying between them. This hypothesis will, of course, be well known to many of our readers, and we have therefore merely noticed it as the doctrine which is adopted by the writer of this essay. Dr. Gellerstedt believes that tubercle may appear under different forms, either as miliary tubercle (the gray semitransparent granulation of authors), or as crude tubercle, nearly, if not quite opaque, and of a yellowish white colour. He does not, however, seem to infer that from either of these two forms we can trace the peculiar appearance termed tubercular infiltration. It will be known to most of our readers, that of late the opinions of the best pathologists have tended to regard this alteration as of inflammatory origin, as being, in fact, the result of a species of scrofulous pneumonia. Dr. Gellerstedt allows that this may possibly be the case, in so far as that pneumonic hepatization may in scrofulous subjects be converted into tubercular hepatization, which in due time may soften, and leave excavations in the lungs.

"But," he adds, "I have never observed such a process to give rise to granular tubercle, except in those cases where the tubercular disease has advanced rapidly with symptoms of pneumonia; and I have then invariably found the tubercular deposit to be of a yellowish white colour, granular, and indistinctly circumscribed, and bearing a strong resemblance to the hepatization and abscesses occurring in the lung after phlebitis."

We have already seen that our author is opposed to the doctrine of the inflammatory origin of tubercle, but he acknowledges that the formation of tubercle and the process of inflammation present many points in common; though, if we trace them carefully, we always find that their mode of development is totally distinct and dissimilar.

The signs of inflammatory action discovered by the microscope in the form of exudation-corpuscles, are easily, he thinks, explained by the fact that tubercles act as a foreign body, and irritate the surrounding parenchyma. The condition of the blood, too, in phthisis, has been much insisted on by the advocates of the inflammation theory; but the cause of the increase of fibrine in that fluid is undoubtedly, he believes, the presence of the tubercular masses, which are already sufficiently far advanced to irritate and inflame the surrounding parenchyma. In fine, Dr. Gellerstedt candidly acknowledges that the influence determining the formation of tubercle in the body has as yet eluded observation; and with other pathologists, he takes refuge in the doctrine of a depression of nervous or vital influence to explain its origin.

In every instance he has found tubercle to occur only as infiltrated into the parenchyma of the various organs wherein it occurred, nor has he ever seen it lying loose in the bronchi, as described by Dr. Carswell.

As to its mode of increase, tubercle has been generally supposed to grow by juxtaposition, and not as in organized bodies by intussusception. Dr. Gellerstedt considers tubercle to be of a low standard of organization, and therefore to be incapable of developing a union between itself and other tissues. The further progress of tubercle from the original cell is produced, he thinks, by the neighbouring blood-vessels, from whence a plasma is thrown out, and this, partly from the influence of the neighbouring tubercular matter, and partly from the peculiar degenerated vitality of the neighbouring tissues, is converted into tubercle. Thus the growth of tubercle bears, he thinks, a close analogy with that of certain other organized products which are destitute of blood-vessels, such as the epithelium, the hair, nails, &c.

In conjunction with Dr. Malmster, who is also, we believe, the author of a recent Essay on Phthisis in Sweden, Dr. Gellerstedt has carried on a series of microscopical observations on the constituents of tubercle, and his conclusions coincide entirely with those of Vogel, in so far as that he never found tubercular matter to consist of anything but an indistinct granular mass, or else of an aggregate of cells; but he did not discover nuclei in the latter. The cells were about the size of $\frac{2}{3}$ of a blood-corpuscle, indistinctly rounded, with ragged edges; and the last seems to be their most constant characteristic. Their contents varied much: sometimes they were filled with a granular matter, at other times they contained many distinct points, and again they occasionally seemed to be entirely empty. Sometimes, too, the cells were numerous; on other occasions they were only thinly dispersed through the granular mass. This appearance of cells was most frequent while the tubercle was as yet gray and semitransparent, but the mass became more amorphous when it had assumed the yellowish-white, opaque aspect, and was then mixed with many small granules, and numerous crystals of cholesterine. Later, when the tubercle was softening, he found it to consist, under the microscope, of a semitransparent fluid, in which floated the above granules and crystals, with globules of fat and also of true pus, and the latter became more numerous as the softening of the tubercular mass advanced.

To the chemical history of tubercle our author has made no additions.

The possibility of the healing of tuberculous disease, he regards as an established fact. He states that tubercle may really be absorbed, but that more frequently it diminishes in size, and becomes surrounded with a cyst, which isolates it from the surrounding parenchyma; at other times it is changed into a calcareous mass.

*Softening of tubercle.* Laennec's idea that tubercle always softened from its centre was, no doubt, formed in accordance with his doctrine of its origin, but Dr. Gellerstedt has always observed them to soften from several points at once, as is also maintained by Dr. Hodgkin. From this last named author, too, he quotes the doctrine, that tubercle preserves its consistence by means of its own special vitality, but when deprived of this essential requisite it rapidly softens and falls away. To this opinion our author assents, as also to that promulgated by Reynaud, that caverns in the lungs may heal in two different ways: in one case, the parenchyma surrounding the cavern becomes indurated, and the bronchi appear as hard white ligaments traversing the indurated portion; in the other form, there is less positive induration of the tissue round the excavation, but it shrinks and draws together, forming a long, firm, white cicatrix. If the pleural surfaces have been previously united by adhesions, these are lengthened and become thinner by the shrinking of the lung, and may perhaps entirely disappear. (?) Dr. Gellerstedt, however, doubts whether all the cicatrized-looking portions at the summit of the lung, are to be referred to the healing of tubercle, and in this doubt we are certain that many of our readers will coincide with ourselves.

The fibrous bands which traverse and surround the pulmonary parenchyma in phthisis, exhibit a similar tendency to contract, and thereby to compress the tissue of the lung, which no doubt, if such, will materially assist the healing of the excavations. The occurrence of these fibrous bands in the lungs is ascribed by Rokitansky to a species of chronic inflammatory action, and they are often observed in connexion with dilatation of the bronchi.

Dr. Gellerstedt fully corroborates the doctrine of Rokitansky and Corrigan, that the dilatation of the bronchi is secondary to, and occasioned by the condensation and contraction of the surrounding tissues. Laennec, it is well known, supported the opposite theory, viz. that the dilatation of the bronchi compressed and condensed the surrounding parenchyma, and Dr. Gellerstedt thinks that this view is not in all cases untenable, as he has occasionally found the bronchi dilated in the lower lobes of the lungs, without any consolidation of the neighbouring tissues.

We regret much to have devoted so short a space to this most interesting portion of Dr. Gellerstedt's essay, but we have throughout endeavoured to pass over, or very briefly notice, those theories already promulgated by other writers, and have confined ourselves as much as possible to the author's original observations. In this, to a certain extent, we feel we do injustice both to Dr. Gellerstedt and to our readers, for much of the value of this essay consists in the judicious arrangement of the opinions of other writers, both those of Sweden and of the other nations of Europe.

The second portion of our author's essay refers to the symptoms of phthisis observed during life; and of these (he remarks) only a few need to be particularly noticed, as they have been from the earliest time the chief objects of attention. Much, however, may still be accomplished in the pursuing of new modes of investigation, and by well-grounded statistical researches into the causes of the malady, and those influences that retard or accelerate its development.

The study of the expectoration our author looks upon as comparatively of small value in phthisis. The most characteristic feature it presents, is the irregular streaked sputum with small whitish granules interspersed. This, however, does not occur till the disease is considerably advanced; at an earlier period he does not think it possible to distinguish the sputa of phthisis from those of common catarrh or bronchitis. Later in the disease, the microscope affords us valuable aid, and is of great benefit in establishing the diagnosis in doubtful cases.

The following are the results of Dr. Gellerstedt's microscopical examination of the sputa: (*a*) Besides the well known presence of a grayish serous looking fluid, in which float numerous epithelial cells with ciliæ and exudation-corpuscles, pus, and mucous globules, none of which are peculiar to phthisis; we find also very minute granules, apparently the nuclei of tubercular cells, but varying greatly in amount. These nuclei are insoluble in ether and in alcohol, but are dissolved by alkalies, and probably consist of albumen. They do not vary either in form or appearance. (*b*) Melanotic matter in the form of small granules, irregularly interspersed or aggregated, (but he has never seen melanotic granules inclosed in cells, as has been described by Bühlmann.) (*c*) Tubercular matter is now recognized by all writers (excepting Gruby) as being found in the expectoration of phthisis when far advanced. It resembles in character that taken from the lungs after death, excepting that the cells so constantly observed in the latter case, are rarely present, and the masses usually consist of aggregated granules, as described by Vogel. The peculiar spherical bodies with concentric rings, discovered by Gruby, and by him regarded as characteristic of phthisis, are, according to Dr. Malmsten, nothing more than *starch-cells* resulting from the diet of the Viennese hospitals, where Gruby's observations were carried on. The various crystals which occur in the sputa, are considered by Dr. Gellerstedt to be of no pathological importance. From all that has been written on this subject, we must conclude with our author, that the microscopical examination of the sputa can be useful as a means of diagnosis only after tubercle has begun to soften, and to be expectorated from the lungs.

*Duration of the disease.* The results of the tables here given, exhibits a much shorter duration of the malady than is allowed by Louis, or by Bayle, or Clark; but we must remember that Dr. Gellerstedt's observations were made upon the soldiers in the garrison, and that in many of these the disorder had perhaps silently advanced for some time before their entrance into the hospital. The average duration of phthisis in Dr. Gellerstedt's patients was not more than four months and eight days; and the mortality of six months gives 88 per cent. Our author observes that perhaps phthisis is really more rapid in Sweden and among the Swedish soldiers, than in France or England; but he tells us, on the other hand, that many of the men persevere in their duties to thc last, and enter the hospital only to die. The length of previous service as a soldier did not appear to retard or hasten the progress of the malady; but the influence of season of the year is undoubted, as has been already ably illustrated by Sir James Clark and others. From want of space, we have abstained from giving any of the valuable tables interspersed in this essay, but the short one below we venture to insert as it exhibits considerable diversity from that of the eminent author just quoted; in reference to the seasons of the year when the mortality from phthisis is highest.

| Sir J. Clark. | Dr. Gellerstedt. | Mortality of each month. Dr. Gellerstedt. |
|---|---|---|
| March | June | 19 |
| February | May | 16 |
| December | October | 14 |
| January | November | 14 |
| April | April | 10 |
| May | March | 8 |
| November | February | 7 |
| June | July | 7 |
| October | August | 7 |
| July | January | 6 |
| September | December | 6 |
| August | September | 5 |

It will be seen from this table that the latter months of spring, and the autumnal season, are the most perilous in Sweden for phthisical patients; but we must ever remember that death does not always occur in those months which have exercised the most injurious influence on the health of of the patient.

*Treatment.* Our author expresses himself very cautiously in respect to the *cure* of phthisis. He allows, however, that cases certainly do occur where every symptom of tubercular disease exists, but the patients nevertheless recover their original health. On the one side, we cannot as yet rely with positive certainty on the symptoms indicating phthisis in the living subject, much as the diagnosis has been improved by auscultation, percussion, and the microscope; and on the other, the appearances in the lungs after death may be fallacious, as to the previous existence and subsequent cure of tubercular disease. In many cases the external symptoms will cease for a time, contemporaneously with diminution of secretion from the caverns, and these last we have seen, Dr. Gellerstedt thinks may really heal, but there is ever danger to the patient, unless the tubercular diathesis be entirely removed. Have we, as yet, discovered the means of effecting this most desirable object?

In general Dr. Gellerstedt has found both lungs affected in those who have died of phthisis, and he has also remarked, that the instances where only one lung was the seat of disease, were precisely those where the progress of the malady was slowest, and where Nature exhibited the strongest tendency to effect a cure. Dr. Gellerstedt does not even deny the possibility of the complete reabsorption of the tubercle:

"I do not consider tubercular matter to be so totally dissimilar in character to those other abnormal tissues which form in the body, and which we know are frequently reabsorbed, as to exclude the possibility of tubercle being acted on in like manner. In spite, however, of the recent wonderful improvements in diagnosis, I do not consider that our means of ascertaining the existence of this disease are so perfect, as that no mistake can be committed regarding the real nature of the malady. Thus we may be easily led to regard as tubercular phthisis, a consolidation of the parenchyma and interstitial cellular substance of the lungs, the result of chronic inflammation, and the more so as this disease, like tubercle, has its seat in the upper lobes, and is often accompanied with dilatation of the bronchi, giving rise to sounds closely resembling those heard in true phthisis."

*Causes of phthisis.* The great mistake of most writers on this point has been, as Dr. Gellerstedt believes, the constant endeavour to find out some special and uniform influence to originate the malady. We allow that too many authors have fallen into this common error, but we still maintain, that, by the highest authorities, it is acknowledged that the most various and opposite causes may here produce the same effect. We shall briefly analyse our author's opinions on this important point:

*a. Climate.* That the climate of Sweden, from its position so near the polar regions, from its vast forests and numerous lakes, would be unfavorable to phthisical constitutions, will naturally be expected; but we were scarcely prepared to find so large a mortality from this disease among the troops in Stockholm. The highest average of deaths from this malady in our army, is that among the black regiments in the West Indies, viz. 14 per thousand, and in the Swedish army at Stockholm, we have an actual mortality from phthisis of 8 per thousand, and an average of 6 more per thousand who are yearly dismissed, at their own request, to die at their own homes. The provinces of Sweden which have, during the last five years, afforded the greatest number of cases of phthisis in the garrison of

Stockholm, are West and North Bothnia, Westmannaland, and Skåne, and the Dales (Dalarna). A carefully-constructed table of these is given by Dr. Gellerstedt, specifying the number of soldiers from each province, their age, &c., but he acknowledges that its value is only approximative.

*b. Trades and professions.* Our author's observations in regard to these are naturally limited, but he considers the life of a soldier, for many reasons, to predispose to phthisis. By an ingeniously constructed table, he attempts to show in one view the trades in which the men were employed previously to entering the service, as also the duration of their military life, and the period of death from tubercular disease.

*c. Age.* The results in respect of age completely coincide with the statements of Sir James Clark, and with the general opinion of medical writers, from Hippocrates to the present time. The highest mortality among the soldiers at Stockholm was between the ages of 20 and 40, being not less than 80 per cent., but of course we must remember that the majority of the military are in service during that period of their lives.

Having thus briefly recorded our author's opinions and experience in regard to the more general causes of phthisis, we will now glance at those which he considers of a more immediate character, and these he divides into—1, such as tend to excite and develop a cachexia already existing but still dormant in the system; and, 2, those which directly favour the outbreak of phthisis.

*a. Hereditary predisposition* is fully acknowledged by Dr. Gellerstedt, who, however, adds nothing new in this respect.

*b. Constitution and temperament.* Dr. Gellerstedt admits to the full extent the existence of the two varieties of habitus phthisicus described by Clark and others, and he further remarks, that among the soldiers of the garrison, a decided predisposition to phthisis was observed in the tallest men.

*c. Mode of life.* Many conditions of a soldier's life have probably an unfavorable influence on his health, and assist in the development of phthisis, but it is difficult to fix on any one circumstance from which these evil results can be said to arise. The barracks inhabited by the Swedish soldiers are in general lofty and well ventilated, excepting those of the 2d Royal Life Guards, but it is precisely in this regiment that the fewest cases of phthisis have occurred during the last five years. Our author very properly calls in question the opinion of Baudelocque, that contaminated air is the chief cause of tubercular disease, and shows that phthisis is very frequent in many provinces of Sweden where the inhabitants spend the whole day in the open air.

*d. The food, clothing, and exercise* of the Swedish soldiers leave, according to Dr. Gellerstedt, nothing to be desired.

*e. Venereal excess,* he observes, does not seem to have any great influence on the development of the malady, but combined with the abuse of alcoholic fluids, we often find that thereby the cachectic condition is produced, which ends in tubercular phthisis. Drunkenness does not seem to prevail to any very great extent in the Swedish army; in the course of five years, 310 cases of delirium tremens have occurred in the garrison of 3000 men, and of these so affected 11 afterwards died of phthisis.

*f. Preceding maladies* undoubtedly predispose a patient to phthisis, by deteriorating the constitution. Dr. Gellerstedt doubts the accuracy of

Vetter's assertion, that gout and hemorrhoids may give rise *directly* to phthisis, though these two maladies, with their congener dyspepsia, often reduce the constitution to that cachectic condition, which is eminently favorable to the development of tubercular disease. In this part of our author's essay an ingenious tabular view is given of the amount of previous disease of all kinds in 119 patients, who ultimately died of phthisis. From this it appears that 44 out of 119 had previous hemoptysis, and 42 had bronchitis, and that the two maladies above named frequently occurred in the same individual. Bronchitis is considered by our author to be a frequent cause of the rapid development of phthisis in those already labouring under the tubercular diathesis, and he believes that acute affections are at all times peculiarly perilous to such constitutions.

*Hemoptysis* he regards as a symptom, and not as a cause of the tubercular disease; he has never met with it among the military without its being accompanied with more or less distinct evidence of the existence of tubercle in the lungs. *Pneumonia* is not, according to our author, a direct excitant of phthisis, but with Rokitansky and others, he believes that the hepatized lung, instead of returning to its natural condition, occasionally becomes the seat of tubercular deposit, and, moreover, that repeated attacks of pneumonia will hasten the development of tubercular cachexia. *Pleurisy* is regarded by Dr. Gellerstedt in the same light; the effusion induces a depressed vitality of the system, by mechanically obstructing the respiration, and thus favours the deposition of tubercle in the lung, but not necessarily in that portion of the pulmonary tissue which has been subjected to pressure from the accumulated fluid. In the same manner he explains the effects of fevers and dyspepsia in producing phthisis, though he allows that the researches of Todd and others have greatly benefited the early history of the disease, by pointing out the influence of dyspepsia in producing a cachectic condition of the system. Is not the dyspepsia, that seems to produce phthisis, sometimes a consequence of the debility of the digestive organs arising from an already existing tubercular diathesis? We are somewhat surprised to find that our author entertains opinions totally opposed to those of Boudet and others, in regard to the influence of intermittent fever upon tubercular disease. So far from believing that ague gives any immunity from phthisis, our author expressly regards it, from its depressing influence on the organs of nutrition, as one of the most cognizable causes of this disease. Perhaps the effects of ague under the climate of Sweden may be different from its agency under the milder sky of Algeria, where M. Boudet's investigations were principally made. But we are already exceeding our limits, and, in conclusion, we can only find space to present our readers with the short *general* table of antecedent diseases given by Dr. Gellerstedt:

| | In 119 Cases. | Per cent. | In 191 Cases. | Per cent. | In 310 Cases. | Per cent. |
|---|---|---|---|---|---|---|
| Bronchitis | 42 | 35·29 | 37 | 19·37 | 79 | 25·48 |
| Hemoptysis | 44 | 36·97 | 84 | 43·97 | 128 | 41·29 |
| Pneumonia | 22 | 18·48 | 51 | 26·70 | 73 | 23·54 |
| Pleurisy | 17 | 14·28 | 28 | 14·65 | 45 | 14·51 |
| Nervous fevers | 10 | 8·40 | 12 | 6·02 | 22 | 7·09 |
| Ague | 27 | 23·52 | 49 | 25·65 | 76 | 24·51 |
| Dyspeptic symptoms | 10 | 8·40 | 32 | 6·02 | 22 | 7·09 |
| Diarrhea and Dysentery | 23 | 19 32 | 33 | 17·27 | 56 | 18·06 |

The figures in the first, third, and fifth columns denote the number of individuals who had suffered from these antecedent maladies, first of those 119 who died of phthisis, then among 191 persons affected with this disease, and lastly among these two classes conjointly, while the per centage of each affection is given in the second, fourth, and sixth columns.

We would gladly have extended our analysis of this interesting essay, but we trust that enough has been here detailed to satisfy our readers that the productions of the Swedish school are entitled to our respect and attention; and that, while our learned brethren of Scandanavia emulate the best British and French writers in attentive personal investigation, they are inferior to none in their acquaintance with the progress of medical science throughout the world.

